# Supporting a sugar tax in New Zealand: Sugar sweetened beverage (‘fizzy drink’) consumption as a normal behaviour within the obesogenic environment

**DOI:** 10.7717/peerj.5821

**Published:** 2018-10-19

**Authors:** Kirsten Robertson, Maree Thyne, James A. Green

**Affiliations:** 1 Department of Marketing, University of Otago, Dunedin, New Zealand; 2 School of Pharmacy, University of Otago, Dunedin, New Zealand; 3 School of Allied Health, University of Limerick, Limerick, Ireland; 4 Health Research Institute (HRI), University of Limerick, Limerick, Ireland

**Keywords:** Obesity, Sugar, Soft drink, Fizzy drink, Healthy eating, Dietary control, Sugar sweetened beverage

## Abstract

**Background:**

Excessive intake of sugar sweetened beverages (SSBs) is a preventable cause of death. While some countries have implemented a tax on SSBs, other countries, such as New Zealand, rely on industry self-regulation and individual responsibility, such as referring to labels, to control one’s own sugar intake from SSBs. The present study examines whether SSB consumers consciously control their diet and therefore interventions such as better labelling might be effective, or alternatively, whether SSB consumers engage in a general pattern of unhealthy eating, and in which case government regulation would be advisable.

**Aim:**

To explore self-reported dietary consumption and conscious healthy eating behaviours of New Zealand consumers who had consumed SSBs over a 24 hour period.

**Method:**

A cross-sectional survey of a representative sample of 2007 New Zealanders, measuring their food and beverage intake over a 24 hour period and self-reported intentions to eat healthily. Within this was a measurement of SSB consumption in the 24 hour period.

**Results:**

Multivariable logistic regression revealed that compared to non-SSB consumers, SSB consumers were more likely to have eaten the following: confectionery; fast food; pre-prepared food; biscuits, cakes or pastries; takeaways; ice-cream/dessert. SSB consumption was also associated with a lower likelihood of referring to food labels, less conscious effort to eat healthily, and to less likely to avoid: sugar; fat; calories; food additives; pre-prepared food. SSB consumers were also less likely to have eaten breakfast, or made a meal at home made from scratch.

**Conclusion:**

SSB consumers were more likely than non-SSB consumers to demonstrate a general pattern of unhealthy eating and were less likely to report consciously controlling their diet. The findings raise significant concerns regarding the efficacy of individual and industry self-regulation and lend support to stronger government targeted interventions.

## Introduction

Excessive consumption of sugar sweetened beverages (SSBs) is a preventable cause of death. They provide little nutritional benefit and are a leading cause of non-communicable diseases (Beaglehole, 2014), including obesity (Malik et al., 2013), type 2 diabetes, risk of cardiovascular disease (Malik et al., 2010), and dental caries (Armfield et al., 2013). Researchers worldwide are advocating for measures to reduce the consumption of SSBs (Backholer et al., 2016; Beaglehole, 2014; Brownell et al., 2009; Singh et al., 2015). While a number of countries have implemented national taxes on SSBs, for instance, Fiji, Spain, Mexico, France, Tonga, Belgium, Saudi Arabia (Thow et al., 2018), other countries such as New Zealand still rely on industry self-regulation. However, research shows self-regulation is not working: the sugar content and serving size of SSBs in countries adopting self-regulation exceed World Health Organisation (WHO) recommendations and New Zealand had the highest sugar content and availability of SSBs in the countries examined (Chepulis et al., 2018).

A tax on SSBs has been called for in New Zealand by the New Zealand Medical Association (2014) and the New Zealand beverage guidance panel (New Zealand Beverage Guidance Panel, 2014), based on observational research that following the implementation of the sugar tax in Mexico, purchases of taxed beverages decreased, while the purchase of untaxed beverages increased (Colchero et al., 2016). Further, leading academic researchers have found the New Zealand public are supportive of a sugar tax, especially if the funds raised by the tax are targeted towards additional programs aimed at addressing obesity (Sundborn et al., 2015). Arguably, a tax on SSBs in New Zealand would have a significant positive impact on population health (Ni Mhurchu et al., 2014). It is predicted that a tax could influence sugar consumption by working both up- and down-stream. Consumption would be lowered by encouraging industry to reduce the sugar content in current products, develop and promote lower sugar content beverages, and by passing on costs of the tax to consumers (New Zealand Beverage Guidance Panel, 2014). How the industry will respond to a sugar tax is uncertain. However, anecdotal evidence from Great Britain suggests that the industry might respond favourably. Prior to the sugar tax introduced in the UK on April 1st 2018, the UK’s soft drink industry reformulated their products to reduce the sugar content (HM Treasury, 2017). For instance, Coca-Cola Great Britain committed to reducing the sugar and calorie content of their products and to investing in other products with less sugar, with the goal to make Great Britain Coca-Cola the first country where 50% of their products are no- or low-calories (Coca-Cola UK, 2015). Despite fears that a sugar tax will negatively impact the industry, media releases in Great Britain show that a recent reduction in sugar content has had little to no effect on sales (Grierson, 2017).

The decision to implement taxes is embroiled within the wider political and business environment. The New Zealand government has set a firm goal to reduce sugar content across the board; however they want to work with industry to set firm expectations before turning to regulation (Jones, 2017), and have suggested measures such as better labelling (Nyika, 2018). ‘Big Businesses’ in New Zealand oppose the tax, arguing that food taxes are an act of a ‘Nanny State’, and encroach on consumer freedom to make their own choices (Jeram, 2016). Researchers point out, however, that the food and media industry have created an obesogenic (obesity promoting) environment in which energy dense, processed, and inexpensive foods have made unhealthy choices the default choice (and obesity the outcome) of people responding normally within the obesogenic environment (Swinburn & Egger, 2004; Swinburn et al., 2011). It is argued that the obesogenic environment has changed weight control from being instinctual to requiring significant cognitive effort—and those who do not consciously control their weight are likely to gain weight (Peters et al., 2002). Indeed, research from the United States suggests SSB consumption does occur within an obesogenic environment with consumption of SSBs linked to poorer diet quality (Piernas et al., 2013). Thus, the question that arises is whether SSB consumers in New Zealand consciously control their diet and therefore interventions such as better labelling, as recommended by the New Zealand government, might be effective. Or rather, whether SSB consumers are a product of the obesogenic environment and engage in a general pattern of unhealthy eating, and in which case government regulation would be necessary (Swinburn et al., 2011).

Using a nationally representative sample, the present study explores (1) associations between SSB consumption and the consumption of healthy and unhealthy food, and (2) whether SSB consumers consciously control their diet by examining SSB consumption and self-reported intentions to eat healthily. Income was included as a proxy for socioeconomic status because dietary intake has been found to be more adverse in lower socioeconomic groups (Metcalf, Scragg & Davis, 2006). Age was included as it has been found to be associated with the consumption of SSBs (University of Otago and Ministry of Health, 2011a).

## Methods

### Study setting

New Zealand has an obesity epidemic. The 2016 figures from the Organisation for Economic Co-operation and Development (OECD) reported 66.8% of the New Zealand population (>15 years) was overweight or obese, placing New Zealand as the third most overweight nation in the OECD Organisation for Economic Co-operation and Development (2016). New Zealanders consume approximately 37 teaspoons of sugar a day (Beaglehole, 2014). This is in stark contrast to World Health Organisation (WHO) recommendations of no more than 12 teaspoons of sugar per day for an average adult (WHO, 2015). Further, 17% of New Zealand adults total sugar intake comes from SSBs (University of Otago and Ministry of Health, 2011a).

### Sample

The data comes from the New Zealand Lifestyles Study—a national study into consumer lifestyles, regularly undertaken by the University of Otago since 1979. Data for this round was collected by Research Now, a leading online market research company operating in 41 countries with over 11 million panellists. Panellists are rewarded for taking part in surveys through a structured incentive scheme, determined by the length of the survey. The New Zealand panel comprises 160,000 active members (defined as having taken part in a survey in the past 12 months). The structured incentives offered in New Zealand include a variety of gift cards. Sampling was customised using targeting criteria, following which email invites were automatically randomised to avoid bias, and staggered to avoid respondents receiving them on the same day. The current sample was a nationally representative sample of 2007 New Zealand citizens. Stratified random sampling was used based on New Zealand census data to ensure the sample was representative of New Zealand. Quotas were age, sex, ethnicity, education and income. The sample ranged in age from 18 to 89 years (see Table 1). The full online survey took approximately 40 min to complete and involved answering 600 questions about consumer opinions, attitudes and behaviour. Only data pertinent to the current study are presented in this paper. This study had ethical approval from the Department of Marketing, under delegated authority for low risk studies from the University of Otago Human Ethics Committee.

### Procedure

*Age, income, employment status and education:* Age was measured as a continuous variable and all other demographic variables were assessed using census categories.

*SSB and Food consumption*: Individuals were asked to indicate whether or not (Yes / No) they had consumed SSBs and engaged in seven less healthy, and four more healthy, food consumption behaviours (adapted from Food Standards Authority UK, 2007 (Food Standards Agency, 2007); see Table 2) in the previous 24 hours.

**Table 1:** Percentage and number of participants consuming SSBs within a 24 hour period as a function of income, age, employment status, and ethnicity.

	Consumed SSBs % (n)	Did not consume% (n)	O.R.(95% CI)
Income			
<20,000	30.0% (57)	70.0% (133)	1.0 (Reference)
20–29,999	28.1% (66)	71.9% (169)	0.9 (0.6, 1.4)
30–39,999	30.5% (67)	69.5% (153)	1.0 (0.7, 1.6)
40–49,999	22.3% (47)	77.7% (164)	0.7 (0.4, 1)
50–59,999	23.1% (67)	64.9% (124)	1.3 (0.8, 1.9)
60–69,999	31.6% (54)	68.4% (124)	1.1 (0.7, 1.7)
70–79,999	28.4% (46)	71.6% (117)	0.9 (0.6, 1.5)
80–89,999	31.8% (35)	68.2% (75)	1.1 (0.7, 1.8)
90–99,999	41.4% (41)	58.6% (58)	1.6 (1, 2.7)
100–109,999	31.6% (42)	68.4% (91)	1.1 (0.7, 1.7)
110–119,999	26.6% (17)	73.4% (47)	0.8 (0.4, 1.6)
120–129,999	34.6% (18)	65.4% (34)	1.2 (0.6, 2.4)
130–139,999	41.2% (14)	58.8% (20)	1.6 (0.8, 3.5)
140–149,999	37.1% (13)	62.9% (22)	1.4 (0.7, 2.9)
Over 150,000	29% (29)	71.0% (71)	1.0 (0.6, 1.6)
Age in years			
18–30	37.2% (191)	62.7% (321)	1.0 (Reference)
31–50	35.4% (256)	64.6% (468)	0.9 (0.7, 1.2)
51–70	23.7% (144)	76.3% (463)	**0.5 (0.4, 0.7)**
71 +	13.4% (22)	86.6% (164)	**0.3 (0.2, 0.4)**
Employment			
Working fulltime for someone else	36.1% (247)	63.9% (437)	1.0 (Reference)
Working part-time for someone else	27.8% (76)	72.2% (197)	**0.7 (0.5, 0.9)**
Self-employed	24.8% (41)	75.2% (124)	**0.6 (0.4, 0.9)**
Unemployed	35.4% (51)	64.6% (93)	1.0 (0.7, 1.4)
Retired	16.9% (57)	83.1% (280)	**0.4 (0.3, 0.5)**
Student	36.7% (83)	63.3% (143)	1.0 (0.8, 1.4)
Homemaker	32.6% (58)	67.4% (120)	0.9 (0.6, 1.2)
Ethnicity			
New Zealand European	30.4% (382)	69.6% (873)	1.0 (Reference)
Māori	41.7% (90)	58.3% (126)	**1.6 (1.2, 2.2)**
Samoan/Cook Island/Tongan/Niuean	32.8% (22)	67.2% (45)	1.1 (0.7, 1.9)
Chinese	22.9% (16)	77.1% (54)	0.7 (0.4, 1.2)
Indian	27.3% (21)	72.7% (56)	0.9 (0.5, 1.4)
Other	25.5% (82)	74.5% (240)	0.8 (0.6, 1)

**Notes.**

Bold values indicate *p* < .05.

*Intention to eat healthily*: Intentional healthy eating behaviours were measured through seven items asking whether respondents avoided specific foods and additives, and one item asking whether they make a conscious effort to eat healthy (adapted from Kähkönen, Tuorila & Rita, 1996). Participants were also asked to indicate their agreement with the statement that they refer to labels to select the most nutritious food (see Table 2). Responses were made on a five-point scale where 1 = ‘strongly disagree’ and 5 = ‘strongly agree’.

## Results

*Description of consumption of SSBs as a function of demographics*: In the present study 30.5% (*n* = 613) of the sample had consumed SSBs in the past 24 hours, including 10.5% who had consumed two or more SSBs during the time period. Males (31.9%) and females (29.2%) were similarly likely to have consumed SSBs (*χ*^2^ = 1.8, *df* = 1, *p* = 0.2). The prevalence of consumption reported in the present study (30.5% in the past 24 h) is similar but higher than findings reported in the New Zealand Adult Nutrition Study which found 23.7% of the adult population consumes soft drinks and / or energy drinks, three or more times a week (University of Otago and Ministry of Health, 2011a). However, there is a differing focus of measurement with the nutrition study combining soft drink and energy drink consumption together, and a different recall time-frame. Descriptive statistics for SSB consumption as a function of age, employment status, education, and household income (see Table 1) revealed that although there was some variation, SSB consumption occurs across the population. SSB consumption decreased with increasing age, and those working part time, self-employed or retired were also less likely to have consumed SSBs in the last 24 hours. Although not the focus of this research, we observed higher consumption of SSBs by Māori, the indigenous people of New Zealand and an ethnic minority, a pattern previously observed in Māori females (University of Otago and Ministry of Health, 2011b). The staggered sampling resulted in a relatively even spread for survey completion across the week, with aggregated data showing participation spread over days of the week, somewhat favouring Monday to Thursday.

**Table 2:** Relationship between 24 hour SSB consumption and other food consumption behaviours.

	**Consumed SSBs**		**Simple**^a^	**Multiple**^b^
	**% Yes**	**% No**	**O.R.[95% CI]**	**O.R.[95% CI]**
**Less Healthy Eating**				
Snacked in between meals	75.5	67.1	**1.5 [1.2, 1.9]**	**1.4 [1.2, 1.8]**
Eaten confectionary (i.e., lollies, potato chips)	71.9	54.6	**2.1 [1.7, 2.6]**	**2.0 [1.7, 2.5]**
Eaten fast-food (i.e., McDonalds)	36.5	11.1	**4.6 [3.6, 5.8]**	**4.3 [3.4, 5.4]**
Eaten takeaways (i.e., Indian, Thai)	17.6	5.7	**3.5 [2.6, 4.8]**	**3.2 [2.3, 4.3]**
Eaten biscuits, cakes, or pastries	52.2	49.4	1.1 [0.9, 1.4]	**1.3 [1.1, 1.6]**
Eaten dessert or ice cream	38.2	27.7	**1.6 [1.3, 2.0]**	**1.8 [1.4, 2.2]**
Eaten a meal at home made from pre-prepared food / sauces	35.7	23.8	**1.8 [1.4, 2.2]**	**1.6 [1.3, 2.0]**
**More Healthy Eating**				
Eaten breakfast	73.4	82.7	**0.6 [0.5, 0.7]**	**0.6 [0.5, 0.8]**
Eaten vegetables	84.5	89	**0.7 [0.5, 0.9]**	0.8 [0.6, 1.0]
Eaten fruit	72.6	78.1	**0.7 [0.6, 0.9]**	0.8 [0.7, 1.0]
Eaten a meal at home that was made from scratch	70.3	79	**0.6 [0.5, 0.8]**	**0.7 [0.6, 0.9]**

**Notes.**

Bold values indicate *p* < .05.

a Simple logistic regression models use SSB consumption in the last 24 hours as binary predictor (1–consumed; 0–not consumed) of each food consumption behaviour (1–consumed last 24 hours; 0–not consumed).

b Multiple predictor models use SSB consumption in the last 24 hours as binary predictor (1–consumed; 0–not consumed) of each food consumption behaviour (1–consumed last 24 hours; 0–not consumed), while controlling for age and income (both treated as continuous predictors).

### Analysis

To explore the relationship between consuming SSBs and food consumption behaviour, simple logistic regressions were conducted with SSB serving as a predictor for all other food consumption behaviours. These were followed by multiple predictor models with SSB consumption, age and income entered as predictors (Table 2). Full details of the models are at available at http://osf.io/vcqw4, along with the raw data. SSB consumers were more likely to eat unhealthy food (e.g., snacks; confectionery; fast food; takeaways; desert or ice-cream; biscuits, cakes or pastries; pre-prepared food), and were less likely to eat healthy food (breakfast or a meal made from scratch). Similar linear regressions were conducted for each healthy eating intention, with SSB consumption predicting each intention, and then in a multiple regression model, controlling for age and income (Table 3). Compared to non SSB consumers, SSB consumers were less likely to check food labels, consciously try to eat healthily, avoid pre-prepared food or foods high in: fat; sugar; calories or additives.

**Table 3:** Relationship between 24 hour SSB consumption and intentions to eat healthily.

	**Consumed SSBs**		**Simple**^a^	**Multiple**^b^
	**Yes(Mean)**	**No(Mean)**	**B[95% CI]**	**B[95% CI]**
Use labels to select nutritious food	3.0	3.3	**−0.31 [−0.41, −0.20]**	**−0.32 [−0.43, −0.22]**
Make a conscious effort to eat healthy	3.5	3.8	**−0.31 [−0.40, −0.23]**	**−0.29 [−0.37, −0.20]**
Make a conscious effort to avoid salt	2.9	3.1	**−0.16 [−0.27, −0.05]**	−0.11 [−0.22, 0.00]
Make a conscious effort to avoid fat	3.1	3.3	**−0.26 [−0.36, −0.16]**	**−0.20 [−0.30, −0.10]**
Make a conscious effort to avoid sugar	3.0	3.4	**−0.33 [−0.43, −0.22]**	**−0.28 [−0.38, −0.17]**
Make a conscious effort to control the number of calories	2.7	2.8	**−0.14 [−0.25, −0.03]**	**−0.12 [−0.23, −0.02]**
Make a conscious effort to avoid food additives	2.7	3.1	**−0.36 [−0.47, −0.26]**	**−0.32 [−0.43, −0.21]**
Make a conscious effort to control my cholesterol	2.9	3.0	**−0.20 [−0.30, −0.08]**	−0.09 [−0.20, 0.02]
Make a conscious effort to avoid pre-prepared food	2.9	3.3	**−0.39 [−0.50, −0.28]**	**−0.37 [−0.48, −0.26]**

**Notes.**

Bold values indicate *p* < .05.

a Simple logistic regression models use SSB consumption in the last 24 hours as binary predictor (1–consumed; 0–not consumed) of each intention to eat healthily (scales from 1–5 with higher values indicating stronger agreement.

b Multiple predictor models use SSB consumption in the last 24 hours as binary predictor (1–consumed; 0–not consumed) of each intention to eat healthily (scales from 1–5 with higher values indicating stronger agreement, while controlling for age and income (both treated as continuous predictors).

## Discussion

This study of SSB consumption within a nationally representative sample of New Zealand examined whether SSB consumers consciously control their diet or rather whether SSB is part of a general pattern of unhealthy eating. Our findings clearly show that compared to non-SSB consumers, SSB consumers are more likely to eat an adverse diet and are less likely to make a conscious effort to eat healthily. In particular, SSB consumers in the current study demonstrated a propensity towards convenience foods, such as fast food and takeaways, consumption of which is associated with being overweight (Van der Horst, Brunner & Siegrist, 2011), and is of significant concern in a country that is already the third most overweight nation in the OECD (Organisation for Economic Co-operation and Development, 2016). Arguably, fast food and SSBs complement each other, and their consumption is often related. However, consumption of SSB’s was also associated with consuming a variety of other unhealthy food such as confectionery, dessert or ice cream, biscuits, cakes or pastries, and pre-prepared food. SSB consumers were also less likely to eat beneficial foods such as breakfast, the omission of which is associated with negative health outcomes (Richards & Smith, 2016). Furthermore, SSB consumers were less likely than non SSB consumers to report making a conscious effort to eat healthily, raising significant concerns regarding the efficacy of soft intervention measures, for instance, relying solely on industry self-regulation. Of note, we found no effect of income, however, we did find an effect for age, suggesting that the relationship between SSB consumption and an adverse diet is similar across socio-economic demographics and more pronounced amongst younger individuals.

The current finding that SSB consumers eat a poor quality diet aligns with research from the United States (Piernas et al., 2013). Moreover, the finding that SSB consumers also report making less conscious effort to try to eat healthily extends previous research that has focused on behaviour only. This lack of behavioural intention raises significant concerns regarding the likelihood that SSB consumers will change their behaviour on their own volition. Past research has demonstrated that the sugar content and serving size of SSBs in countries with industry regulation exceed WHO recommendations (Chepulis et al., 2018). In these countries, the onus falls on individuals to regulate the quantity and serving size of sugar they consume from SSBs. However, the present findings show SSB consumers are less likely than others to try to avoid sugar or calories. Thus measures to increase individual responsibility such as better labelling as recommended by the New Zealand government (Nyika, 2018) are unlikely to be effective. In line with researchers across the world (Backholer et al., 2016; Beaglehole, 2014; Brownell et al., 2009; Singh et al., 2015), we advocate for stronger measures to reduce the consumption of SSBs.

The current study had a number of strengths, including the large and representative sample; however, the cross-sectional nature of the data means cause and effect cannot be examined. Longitudinal research is needed to identify the causal relationship. Furthermore, the findings are based on consumer’s self-reported consumption over a 24 hour period and thus different relationships might have been observed if we included a more representative food diary. It should be noted, however, that the use of staggered invitations over a 7-day period, facilitated the collection of food consumption behaviour across a week. However, we did not analyse the results based on the day of the week that participants completed the survey. It is possible that respondents completing the survey in the weekend might have been more likely to have consumed fast food or takeaways, and thus, also been more likely to consume SSBs, as the two often complement each other. However, this explanation does not explain the consumption of the many other unhealthy food types SSB consumers ate, which are arguably consumed any day of the week, nor does it explain their lesser motivations to consciously try to eat healthily. Finally, the use of a stratified quota enabled us to collect responses from a sample matching the characteristics of the New Zealand population used in the quota. However, our quota only matched the New Zealand population in terms of age, sex, ethnicity, education and income, and may not be representative on other characteristics that were not part of the quota. Further, because potential participants self-select into the larger panel from which our sample was recruited, people that opt in to the panel may differ systematically from those who do not.

## Conclusion

Despite the findings being drawn from a cross-sectional study, the finding that SSB consumers eat a less healthy diet and are less likely to consciously try to eat a healthy diet questions the assumption of ‘Big Businesses’, that people make the best decisions they can (Jeram, 2016). Given that the New Zealand public are supportive of a sugar tax (Sundborn et al., 2015), 17% of adults’ total sugar intake comes from SSBs (University of Otago and Ministry of Health, 2011a), and the current findings showing that SSB consumers show limited healthy eating behaviour (or indeed control of sugar intake), we feel a sugar tax is justified. We therefore support the sugar tax recommendation by the New Zealand Medical Association (2014) and the New Zealand beverage guidance panel (New Zealand Beverage Guidance Panel, 2014). The correlation between SSB consumption and other unhealthy food consumption also suggests that an intervention on SSB consumption would need to be supported by a wider intervention, targeting the obesogenic environment.

## Additional Information and Declarations

### Data Availability

The following information was supplied regarding data availability: Anonymized raw data is available at the Open Science Framework: Robertson, K., Thyne, M., & Green, J.A. (2018, August 8). Sugar sweetened beverages. http://osf.io/vcqw4.

## References

[ref-1] Armfield JM, Spencer AJ, Roberts-Thomson KF, Plastow K (2013). Water fluoridation and the association of sugar-sweetened beverage consumption and dental caries in Australian children. American Journal of Public Health.

[ref-2] Backholer K, Sarink D, Beauchamp A, Keating C, Loh V, Ball K, Martin J, Peeters A (2016). The impact of a tax on sugar-sweetened beverages according to socio-economic position: a systematic review of the evidence. Public Health Nutrition.

[ref-3] Beaglehole R (2014). Sugar sweetened beverages, obesity, diabetes and oral health: a preventable crisis. Pacific Health Dialogue.

[ref-4] Brownell KD, Farley T, Willett WC, Popkin BM, Chaloupka FJ, Thompson JW, Ludwig DS (2009). The public health and economic benefits of taxing sugar-sweetened beverages. New England Journal of Medicine.

[ref-5] Chepulis L, Mearns G, Hill S, Wu JH, Crino M, Alderton S, Jenner K (2018). The nutritional content of supermarket beverages: a cross-sectional analysis of New Zealand, Australia, Canada and the UK. Public Health Nutrition.

[ref-6] Coca-Cola UK (2015). Choice and information: delivering out commitments. https://www.coca-cola.co.uk/content/dam/journey/gb/en/hidden/PDFs/coca-cola-choice-and-information-report.pdf.

[ref-7] Colchero MA, Popkin BM, Rivera JA, Ng SW (2016). Beverage purchases from stores in Mexico under the excise tax on sugar sweetened beverages: observational study. BMJ.

[ref-8] Food Standards Agency (2007). Consumer attitudes to food. United Kingdom. http://webarchive.nationalarchives.gov.uk/20111206144033/http://www.food.gov.uk/multimedia/pdfs/cas07uk.pdf.

[ref-9] Grierson J (2017). Coca-Cola says sugar cuts have not harmed sales. The Guardian. https://www.theguardian.com/society/2017/may/17/coca-cola-says-sugar-cuts-have-not-harmed-sales.

[ref-10] HM Treasury (2017). The soft drinks industry levy. https://www.bda.uk.com/professional/influencing/treasury_infosheet_on_sugar_levy.

[ref-11] Jeram J (2016). The health of the state.

[ref-12] Jones N (2017). Food and drink giants told ‘all options are on the table’. http://www.nzherald.co.nz/nz/news/article.cfm?c_id=1&objectid=11938048.

[ref-13] Kähkönen P, Tuorila H, Rita H (1996). How information enhances acceptability of a low-fat spread. Food Quality and Preference.

[ref-14] Malik VS, Pan A, Willett WC, Hu FB (2013). Sugar-sweetened beverages and weight gain in children and adults: a systematic review and meta-analysis. The American Journal of Clinical Nutrition.

[ref-15] Malik VS, Popkin BM, Bray GA, Després JP, Hu FB (2010). Sugar-sweetened beverages, obesity, type 2 diabetes mellitus, and cardiovascular disease risk. Circulation.

[ref-16] Metcalf P, Scragg R, Davis P (2006). Dietary intakes by different markers of socioeconomic status: results of a New Zealand workforce survey. New Zealand Medical Journal.

[ref-17] New Zealand Beverage Guidance Panel (2014). POLICY BRIEF: options to reduce sugar sweetened beverage (SSB) consumption in New Zealand. Pacific Health Dialogue.

[ref-18] New Zealand Medical Association (2014). NZMA policy briefing: tackling obesity.

[ref-19] Ni Mhurchu C, Eyles H, Genc M, Blakely T (2014). Twenty percent tax on fizzy drinks could save lives and generate millions in revenue for health programmes in New Zealand. New Zealand Medical Journal.

[ref-20] Nyika R (2018). Sugary drinks—NZ worse than Canada, UK and Australia, study finds. Stuff News. https://www.stuff.co.nz/national/health/100581810/sugary-drinks--nz-worse-than-usa-uk-and-australia-study-finds.

[ref-21] Organisation for Economic Co-operation and Development (2016). Overweight or obese population. https://data.oecd.org/healthrisk/overweight-or-obese-population.htm.

[ref-22] Peters JC, Wyatt HR, Donahoo WT, Hill JO (2002). From instinct to intellect: the challenge of maintaining healthy weight in the modern world. Obesity Reviews.

[ref-23] Piernas C, Mendez MA, Ng SW, Gordon-Larsen P, Popkin BM (2013). Low-calorie-and calorie-sweetened beverages: diet quality, food intake, and purchase patterns of US household consumers. The American Journal of Clinical Nutrition.

[ref-24] Richards G, Smith AP (2016). Breakfast and energy drink consumption in secondary school children: breakfast omission, in isolation or in combination with frequent energy drink use, is associated with stress, anxiety, and depression cross-sectionally, but not at 6-month follow-up. Frontiers in Psychology.

[ref-25] Singh GM, Micha R, Khatibzadeh S, Lim S, Ezzati M, Mozaffarian D (2015). Estimated global, regional, and national disease burdens related to sugar-sweetened beverage consumption in 2010. Circulation.

[ref-26] Sundborn G, Thornley S, Lang B, Beaglehole R (2015). New Zealand’s growing thirst for a sugar-sweetened beverage tax. New Zealand Medical Journal.

[ref-27] Swinburn B, Egger G (2004). The runaway weight gain train: too many accelerators, not enough brakes. BMJ.

[ref-28] Swinburn BA, Sacks G, Hall KD, McPherson K, Finegood DT, Moodie ML, Gortmaker SL (2011). The global obesity pandemic: shaped by global drivers and local environments. The Lancet.

[ref-29] Thow AM, Downs SM, Mayes C, Trevena H, Waqanivalu T, Cawley J (2018). Fiscal policy to improve diets and prevent noncommunicable diseases: from recommendations to action. Bulletin of the World Health Organization.

[ref-30] University of Otago and Ministry of Health (2011a). A focus on nutrition: key findings of the 2008/09 New Zealand adult nutrition survey.

[ref-31] University of Otago and Ministry of Health (2011b). A focus on Mãori nutrition: key findings of the 2008/09 New Zealand adult nutrition survey.

[ref-32] Van der Horst K, Brunner TA, Siegrist M (2011). Ready-meal consumption: associations with weight status and cooking skills. Public Health Nutrition.

[ref-33] World Health Organization (WHO) (2015). Guideline: sugars intake for adult and children.

